# Negative Mass in the Systems Driven by Entropic Forces

**DOI:** 10.3390/ma18173958

**Published:** 2025-08-24

**Authors:** Edward Bormashenko, Artem Gilevich, Shraga Shoval

**Affiliations:** 1 Department of Chemical Engineering, Biotechnology and Materials, Faculty of Engineering, Ariel University, Ariel 407000, Israel; 2 Department of Industrial Engineering and Management, Faculty of Engineering, Ariel University, P.O. Box 3, Ariel 407000, Israel

**Keywords:** core-shell system, negative mass, negative density, entropic force, polymer spring, temperature dependence

## Abstract

The paper addresses the phenomena of negative effective mass and negative effective density emerging in systems driven by entropic elastic forces. The elasticity of polymers is, at least partially, of entropic origin, and it represents the tendency of a polymer to evolve into a more probable state, rather than into one of lower potential energy. Entropy forces are temperature-dependent; thus, the temperature dependence of the effective mass and effective density arises. The effect of the negative effective mass is a resonance effect, emerging in core–shell mechanical systems, which takes place when the frequency of the harmonic external force acting on a core–shell system connected by an ideal spring approaches from above to the eigen-frequency of the system. We address the situation when the ideal spring connecting the core to the shell is made from a polymer material, and its elasticity is of an entropic origin. The effective mass is calculated, and it is temperature-dependent. The chain of core–shell units connected with a polymer spring is studied. The effective density of the spring is temperature-dependent. Optical and acoustical branches of vibrations are elucidated. The negative mass and density become attainable under the variation of the temperature of the system. In the situation when only one of the springs demonstrates temperature dependence, entropic behavior is investigated. Exemplifications of the effect are addressed.

## 1. Introduction

Entropic forces are emergent forces that arise from the statistical tendency of a system to increase its entropy [1,2]. Unlike other fundamental forces (like gravity or electromagnetism), entropic forces originate from the system’s tendency to maximize the number of accessible microstates [3]. The entropic origin of these forces represents the tendency of a system to evolve into a more probable state, rather than simply into one of lower potential energy [1]. The elasticity of polymers is driven to a great extent by entropy [4,5,6,7]. Maximizing the entropy of a polymer chain implies reducing the distance between its two free ends. Consequently, an entropic elastic force emerges that tends to collapse the chain. Elasticity of the muscles arises from entropy in a way very similar to the entropy-driven elasticity of polymer chains [8]. Entropic forces drive the contraction of cytoskeletal networks [9]. Verlinde suggested that gravity is actually an entropic force [10]. According to Verlinde, gravity emerges from fundamental principles of statistical mechanics and information theory rather than being a fundamental interaction like electromagnetism. His idea is rooted in the holographic principle and thermodynamics [10]. Verlinde showed that if information about matter is stored on a holographic screen (a surface encoding information about space), then the tendency of entropy is to maximize leads in an effect that mimics Newton’s law of gravity [10]. In a similar way, the Coulomb interaction was treated as an entropic force [11].

Usually, entropic forces grow with temperature. However, exceptions to this rule were reported when a system of elementary magnets supposed to be in thermal equilibrium with a thermal bath were exposed to an external magnetic field [12]. A diversity of polymer materials demonstrate entropic elasticity, including rubber [13], Polydimethylsiloxane PDMS [14] and thermoplastic elastomers [15,16]. Entropic elasticity is inherent for tropocollagen, which is the building block of collagen fibrils and fibers that provide mechanical support in connective tissues [17]. Entropic elasticity was observed in slide-ring gels [18]. Somewhat surprisingly, entropic elasticity was reported in cubic crystals of $ScF_{3}$ [19].

Our paper addresses the situation when the entropic/polymer spring gives rise to the effect of the negative mass. The effect of the negative mass is a resonance effect, emerging in core–shell mechanical systems. This effect occurs when the frequency of the harmonic external force acting on a core–shell system, connected by a Hookean massless spring, approaches from above to the eigen-frequency of the system [20,21,22,23,24]. Negative-inertia converters for both translational and rotational motion were introduced [25].

The energy of the vibrated core–shell system is not conserved, due to the fact that it is exposed to the external harmonic force as it occurs, for example, in the famous Kapitza pendulum, in which the pivot point vibrates in a vertical direction, up and down [26,27]. Unlike the Kapitza pendulum, the effect of “negative effective mass” arises in linear approximation to an analysis of the motion [20,21,22,28]. The effect of the negative effective mass/negative effective density may be achieved with the plasma oscillations of free electron gas in metals [29,30,31].

The effects of negative mass and negative density gave rise to the novel mechanic and thermal metamaterials [32,33,34]. The negative effective mass materials were manufactured by the dispersion of soft silicon rubber coated heavy spheres in epoxy, acting as the mechanical resonators [20]. The negative density metamaterial was manufactured in an aluminum plate, comprising the resonant structure [35]. Soft 3D acoustic metamaterials and polymer materials demonstrating negative effective density were reported [36]. Our paper is devoted to the possibility of the realization of negative mass/density metamaterials exploiting entropic elastic forces.

## 2. Materials and Methods

Numerical calculations were performed with Wolfram Mathematica software, version 14.3.

## 3. Results

### 3.1. Negative Mass in the Core–Shell System Driven by Entropic Elastic Force

**Figure 1:**
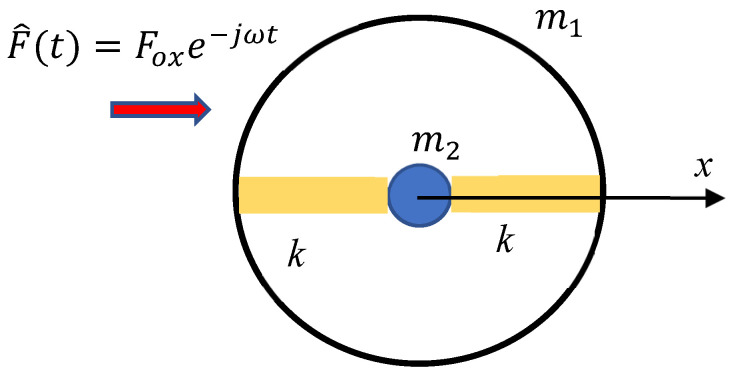
The core–shell unit giving rise to the effect of the “negative effective mass”. The core mass *m* is connected with two polymer elastic Hookean springs *k* to the shell $m_{1}$. The core–shell system is exposed to the harmonic external force $\hat{F}(t)=F_{ox}e^{-j\omega t}$.

Consider the core–shell mechanical system, depicted in Figure 1. The core mass $m_{2}$ is connected to the shell $m_{1}$ with two polymer stripes/springs. The entire system is subjected to the external sinusoidal force $Im(\hat{F}\left(t\right))=F_{0x}sin\omega t$, as shown in Figure 1. We assume that the masses of the polymer stripes are much smaller than both the masses of the core and shell; thus, the masses of the polymer springs are negligible. The core–shell system may be replaced with a single effective mass $m_{eff}$ expressed with Equation (1) (for the rigorous derivation of Equation (1), see [23,24,28,29]):

(1) $$m_{eff}=m_{1}+\frac{m_{2}\omega_{0}^{2}}{\omega_{0}^{2}-\omega^{2}}$$

where $\omega_{0}=\sqrt{\frac{2k}{m_{2}}}$, and *k* is the elastic constant of the polymer stripe (the core mass is driven by the pair of polymer springs).

It is easily seen from Equation (1) that, when the frequency ω approaches $\omega_{0}$ from above, the effective mass $m_{eff}$ will be negative [21,22,28,29]. For the sake of simplicity, we assume that the polymer stripe/spring is built of *ξ* identical polymer chains, each of which may be represented by the ideal Kuhn equivalent freely jointed chain, built of *N* Kuhn monomers; the length of the Kuhn segment is *b* [5]. The elastic constant *k* of the polymer spring is given by Equation (2):

(2) $$k=3\xi \frac{k_{B}T}{Nb^{2}}$$

where $k_{B}$ is the Boltzmann constant, and *T* is the temperature; we assume that the temperature is constant along the addressed core–shell system [5]. Substitution of Equation (2) into Equation (1) yields the following equation for the effective mass of the entire core–shell system (the masses of the polymer springs are neglected):

(3) $$m_{eff}\left(\omega ,T\right)=m_{1}+\frac{6\xi k_{B}T}{Nb^{2}}/\left(\frac{6\xi }{m_{2}}\frac{k_{B}T}{Nb^{2}}-\omega^{2}\right)$$

Now, we fix the frequency of the external force *ω*, and vary the temperature of the core–shell system *T*. It is clearly seen that the effective mass $m_{eff}\left(\omega ,T\right)$ becomes negative when the temperature of the core–shell system approaches the critical temperature $T^{*}$ from below, where $T^{*}$ is given by Equation (4):

(4) $$T^{*}=\frac{m_{2}N{\left(b\omega \right)}^{2}}{6\xi k_{B}}$$

**Figure 2:**
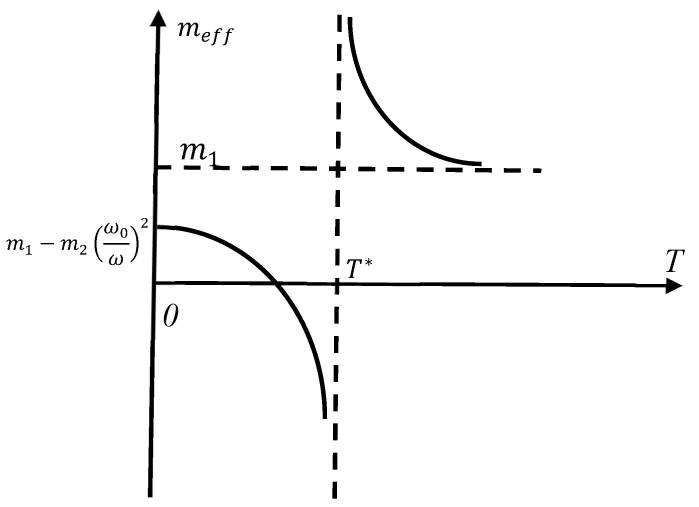
Temperature dependence of the effective mass $m_{eff}\left(T\right)$ is depicted. $T^{*}=\frac{m_{2}N{\left(b\omega \right)}^{2}}{6\xi k_{B}}.$ Dashed lines demonstrate the asymptotic behavior of $m_{eff}\left(T\right)$.

The dependence $m_{eff}(T)$ is presented in Figure 2 (the value of *ω* is fixed). It is instructive to calculate the asymptotic values of $m_{eff}(T)$. When, $T\gg T^{*}$ we derive the following from Equation (3).

(5) $$\underset{T\gg T^{*}}{\lim}m_{eff}\left(T\right)=m_{1}+m_{2}$$

which is intuitively clear for an infinitely stiff spring. The low-temperature limit of the effective mass is also easily calculated as follows:

(6) $$\underset{T\ll T^{*}}{\lim}m_{eff}\left(T\right)=m_{1}$$

Equation (6) is also intuitively clear; indeed, the influence of the “weak” polymer spring (the temperatures are low) becomes negligible.

### 3.2. Negative Density of the Chain of Core–Shell Systems Driven by Elastic Forces

**Figure 3:**
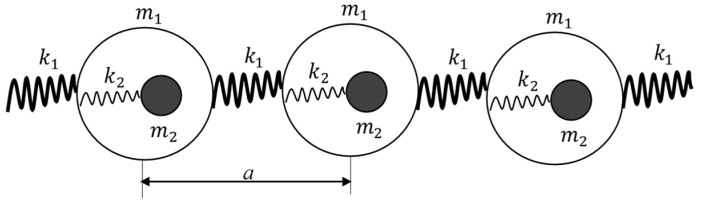
Chain of the core–shell units giving rise to the effect of temperature-dependent negative density [22]. The single lattice constant of the 1D chain, defined as the distance between the core–shell units, is *a*; the mass of the core is $m_{1}$; the mass of the shell is $m_{2}$; $k_{1}$ and $k_{2}$ are entropic strings.

The concept of negative resonant density emerging in a chain of core–shell units, depicted in Figure 3, was introduced in [29]. The effective density of the chain is depicted in Figure 3; $\rho_{eff}\left(\omega \right)$ was calculated in [29], and it is given by Equation (7):

(7) $${\rho_{eff}\left(\omega \right)=\rho }_{st\;}\frac{\theta }{\delta \left(1+\theta \right){\left(\frac{\omega }{\omega_{0}}\right)}^{2}}\;{\left\{{cos}^{-1}\left\{1-\frac{\delta }{2\theta }\frac{{\left(\frac{\omega }{\omega_{0}}\right)}^{2}\left[{\left(\frac{\omega }{\omega_{0}}\right)}^{2}-\left(1+\theta \right)\right]}{{\left(\frac{\omega }{\omega_{0}}\right)}^{2}-1}\right\}\right\}}^{2}$$

where $m_{1}$ and $m_{2}$ are the masses of the shell and core, respectively; the linear density of the chain $\rho_{st}$ is given by $\rho_{st}=\frac{m_{1}+m_{2}}{a}$; $\left[\rho_{st}\right]=\frac{kg}{m}$; $\theta =\frac{m_{2}}{m_{1}}$; $\delta =\frac{k_{2}}{k_{1}}$; *a* is the lattice constant (see Figure 3); and $\omega_{0}=\sqrt{\frac{k_{2}}{m_{2}}}$. It was demonstrated that the effective density becomes negative, when the frequency of the external force *ω* approaches $\omega_{0}$ from above [29].

Now, we assume that both springs are polymer stripes. The elasticity of the stripes is given by Equations (8) and (9) [5]:

(8) $$k_{1}=3\xi_{1}\frac{k_{B}T}{N_{1}b_{1}^{2}}=\alpha_{1}T$$

(9) $$k_{2}=3\xi_{2}\frac{k_{B}T}{N_{2}b_{2}^{2}}=\alpha_{2}T$$

where $\xi_{i},N_{i},b_{i},\;i=1,2$ are the numbers and parameters of the Kuhn chains constituting the strings, $\alpha_{i}=3\xi_{i}\frac{k_{B}}{N_{i}b_{i}^{2}},\;i=1,2$. It is noteworthy that the parameter $\delta =\frac{k_{2}}{k_{1}}$ is temperature-independent. Thus, the squared resonant frequency $\omega_{0}^{2}$ is given by Equation (10):

(10) $$\omega_{0}^{2}=3\xi_{2}\frac{k_{B}T}{{m_{2}N}_{2}b_{2}^{2}}=\frac{\alpha_{2}T}{m_{2}}$$

where $\alpha_{2}=3\xi_{2}\frac{k_{B}}{N_{2}b_{2}^{2}}$. Hence, the effective density of the chain appears as follows:

(11) $${\rho_{eff}\left(\omega ,T\right)=\rho }_{st\;}\frac{\theta }{\delta \left(1+\theta \right)\frac{m_{2}\omega^{2}}{\alpha_{2}T}}\;{\left\{{cos}^{-1}\left\{1-\frac{\delta }{2\theta }\frac{\frac{m_{2}\omega^{2}}{\alpha_{2}T}\left[\frac{m_{2}\omega^{2}}{\alpha_{2}T}-\left(1+\theta \right)\right]}{\frac{{m_{2}\omega }^{2}}{\alpha_{2}T}-1}\right\}\right\}}^{2}$$

**Figure 4:**
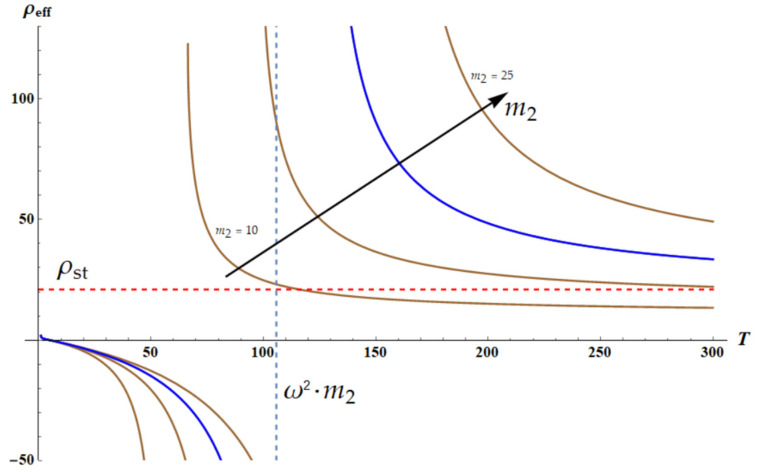
The temperature-dependent effective density of the chain shown in Figure 3 is depicted. The blue curve depicts the dependence $\rho_{eff}\left(\omega ,T\right)$ for the following dimensionless parameters: $k_{1}=k_{2}=1\times T;\;m_{1}=1;\;\delta =1;\;\theta =m_{2};\;a=1;\;\omega =2.3;\;m_{2}=20$. The resonance occurs when $T^{*}=\omega^{2}m_{2}.$ Set of brown curves depicts the temperature dependencies of the effective density calculated for the different values of ${10\leq m}_{2}\leq 25$. Blue dashed line demonstrates the asymptotic behavior of $\rho_{eff}\left(T\right).$ Red dashed line is ${\rho_{eff}=\rho }_{st}=\frac{m_{1}+m_{2}}{a}.$ Black arrow depicts increase in $m_{2}$.

Now, we fix the frequency of the external force *ω*. The plot $\rho_{eff}\left(T\right)$ is depicted in Figure 4. The graph is numerically built with Wolfram Mathematica software. The blue curve depicts the dependence $\rho_{eff}\left(\omega ,T\right)$ for the following dimensionless parameters: $k_{1}=k_{2}=1\times T;\;m_{1}=1;\;\delta =1;\;\theta =m_{2};\;a=1;\;\omega =2.3;\;m_{2}=20$.

It is recognized that $\rho_{eff}\left(T\right)$ becomes negative when the temperature of the core–shell system approaches the critical temperature $T^{*}$ from below, where $T^{*}$ is given by Equation (12):

(12) $$T^{*}=\frac{\omega^{2}m_{2}}{\alpha_{2}}$$

where $\alpha_{2}=3\xi_{2}\frac{k_{B}}{N_{2}b_{2}^{2}}$. The set of brown curves appearing in Figure 4 depict the temperature dependencies of the effective density $\rho_{eff}(T)$ calculated for the different values of ${10\leq m}_{2}\leq 25$. It is clearly demonstrated that the high-temperature limit of the effective density is given by Equation (13):

(13) $$\underset{T\to \infty }{\lim}\rho_{eff}\left(\omega ,T\right)=\rho_{st}=\frac{m_{1}+m_{2}}{a}$$

which is as expected for the massless, infinitely stiff springs.

**Figure 5:**
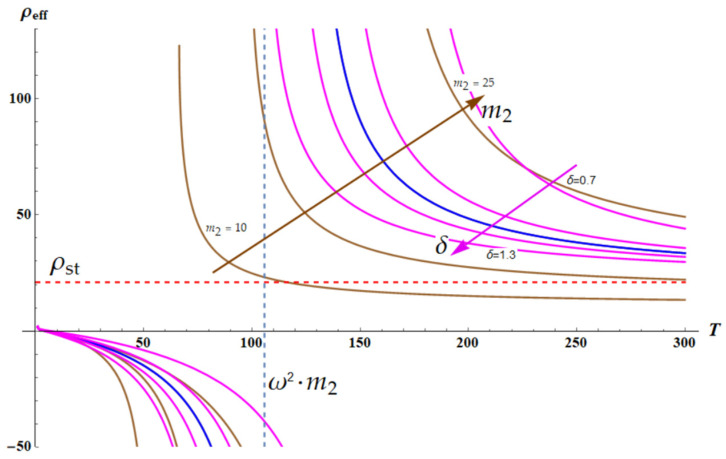
The plots demonstrate changes in the resonance curves when parameters $m_{2}$ and δ are varied. Brown curves are built for a fixed δ=1 and demonstrate a change in $\rho_{eff}$ with ω for ${10\leq m}_{2}\leq 25$. The blue curve demonstrates $\rho_{eff}\left(T;\omega =2.3,m_{2}=20,\;\delta =1\right).$ The magenta curves illustrate variations in 0.7<δ<1.3 and depict $\rho_{eff}\left(T;\omega =2.3,m_{2}=20,\;\delta \in \{0.7,\;0.9,\;1.1,\;1.3\}\right).$ The brown arrow depicts increase in $m_{2}$; the magenta arrow illustrates increase in δ.

Let us vary the parameter δ in Equation (7) in the range less than and greater than one, namely 0.7<δ<1.3. Parameter $\delta =\frac{k_{2}}{k_{1}}$ quantifies the relative stiffness of spring $k_{2}$ in reference to the string $k_{1}.$ The variation of parameters $m_{2}$ and δ is illustrated in Figure 5. Brown curves are built for the fixed δ=1 and demonstrate a change in $\rho_{eff}$ with ω for ${10\leq m}_{2}\leq 25$. Magenta curves, in turn, illustrate a fixed $m_{2}=20$ and 0.7<δ<1.3.

Figure 5 illustrates a very important result: increase in $\delta =\frac{k_{2}}{k_{1}}$ leads to the sharpening of the resonance behavior of $\rho_{eff}(T)$ dependence. This result is intuitively quite understandable; indeed, the decrease in stiffness of the outer spring $k_{1}$ results in the sharpening of the resonance.

**Figure 6:**
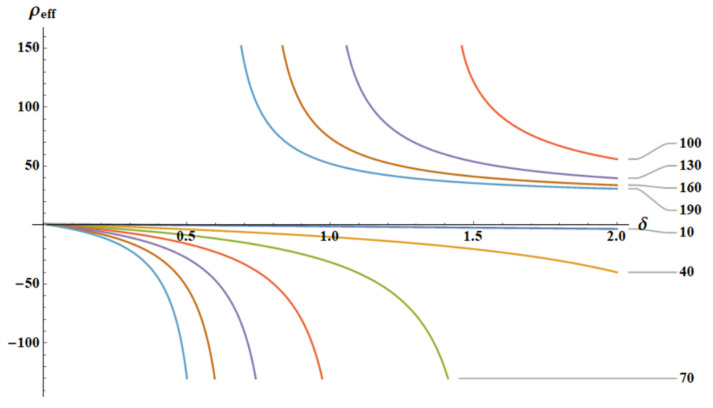
The dependence $\rho_{eff}(\delta )$ is depicted for various temperatures *T*. The curves $\rho_{eff}\left(\delta ;\omega =2.3,m_{2}=20,\;T\in [10,\;190]\right)$ are depicted.

The dependence $\rho_{eff}\left(\delta \right)$ as calculated for different temperatures is depicted in Figure 6. In the low-temperature limit, when $T\ll \omega^{2}m_{2}$ and δ≪1, the effective density $\rho_{eff}\left(T,\;\omega \right)$ almost does not change with $\delta =\frac{k_{2}}{k_{1}}$, as is illustrated in Figure 6.

Now, we address a situation when we remove the temperature dependence of the elasticity of the stripes (Equations (8) and (9)) one by one. In the first case, $k_{1}\left(T\right)=T$ and $k_{2}=const\left(T\right)=1$. Then, Equation (7) with dimensionless parameters will transform into Equation (14):

(14) $$\rho_{eff}\left(T;\omega ,\alpha_{2}\right)=\frac{T}{\omega^{2}}{{cos}^{-1}[1-\frac{\omega^{2}(-1-m_{2}+\omega^{2}m_{2})}{2T(-1+\omega^{2}m_{2})}]}^{2}$$

**Figure 7:**
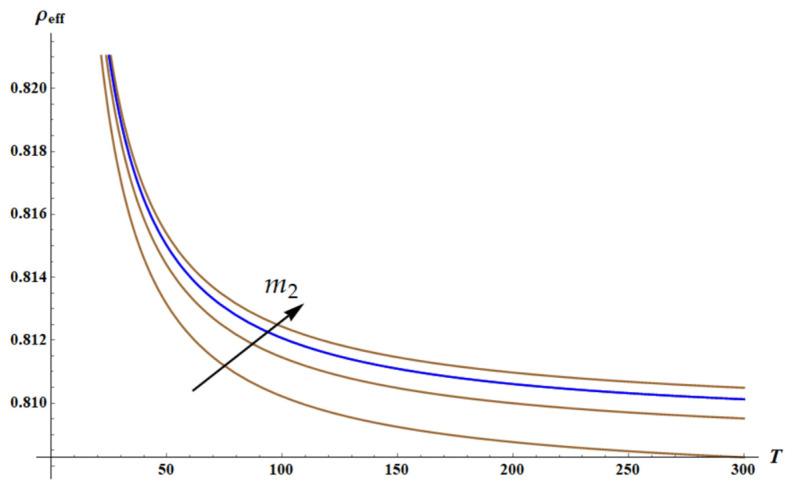
Curves $\rho_{eff}\left({T;m}_{2}\in \left\{10,15,20\left(Blue\right),25\right\},\omega =2.3\right)$ are depicted. Black arrow depicts the increase in $m_{2}$. $\underset{T\to \infty }{\mathrm{Lim}}\left(\rho_{eff}\left(T,\omega \right)\right)=1+\frac{m_{2}}{1-\omega^{2}m_{2}}$.

The dependence $\rho_{eff}\left(T\right)$ calculated for the different values of parameter $m_{2}$ is illustrated in Figure 7.

The second case corresponds to $k_{1}=const\left(T\right)=1,$ $k_{2}(T)=T$. Then, Equation (7) with dimensionless parameters will transform into Equation (15):

(15) $$\rho_{eff}\left(T,\;\omega \right)=\frac{1}{\omega^{2}}{{cos}^{-1}[1-\frac{\omega^{2}(-1-m_{2}+\frac{\omega^{2}m_{2}}{T})}{2(-1+\frac{\omega^{2}m_{2}}{T})}]}^{2}$$

**Figure 8:**
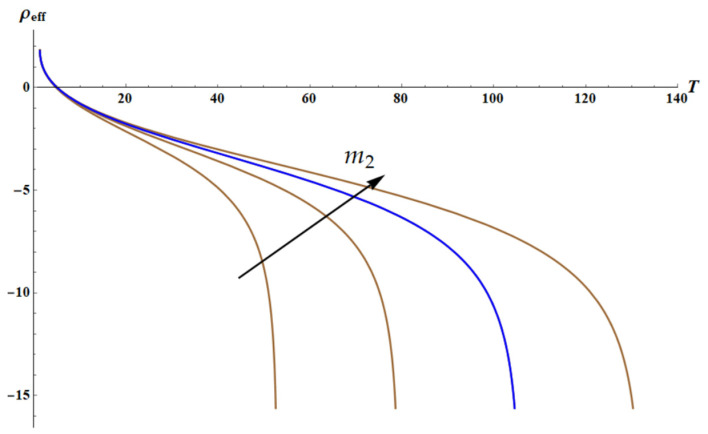
Here are curves $\rho_{eff}\left({T;\;m}_{2}\in \left\{10,\;15,\;20\;\left(Blue\right),\;25\right\},\;\omega =2.3\right)$. The asymptotic behavior corresponds to $T=\omega^{2}m_{2}$. Black arrow depicts the increase in $m_{2}$.

The dependence $\rho_{eff}\left(T\right)$ calculated for the different values of parameter $m_{2}$ is illustrated in Figure 8.

The field of negative densities is clearly recognized in Figure 8.

### 3.3. Dispersion Equations: Influence of the Temperature

The dispersion equation for the 1D lattice (Figure 3) is given by [29,30,31]:

(16) $$m_{1}m_{2}\omega^{4}-\left[\left(m_{1}+m_{2}\right)k_{2}+{2k}_{1}m_{2}\left(1-\cos\left(qa\right)\right)\right]\omega^{2}+{2k}_{1}k_{2}\left(1-\cos\left(qa\right)\right)=0$$

Considering, as earlier, the dimensionless parameters $m_{1}=1,\;\delta =1,\;\theta =m_{2},\;a=1$, Equation (16) yields the following:

(17) $$m_{2}\omega^{4}-\left[\left(1+m_{2}\right)k_{2}+2k_{1}m_{2}\left(1-cos(q\right)\right]\omega^{2}+2k_{1}*k_{2}\left(1-\cos\left(qa\right)\right)=0$$

For $k_{1}(T)=T,$ $k_{2}(T)=T$ (both of springs are entropic), we have the following:

(18) $$\frac{1}{{\left(T/m_{2}\right)}^{2}}\omega^{4}-\left[\left(1+m_{2}\right)+2m_{2}\left(1-cos(q\right)\right]\frac{\omega^{2}}{T/m_{2}}+2m_{2}\left(1-cos(q\right)=0$$

**Figure 9:**
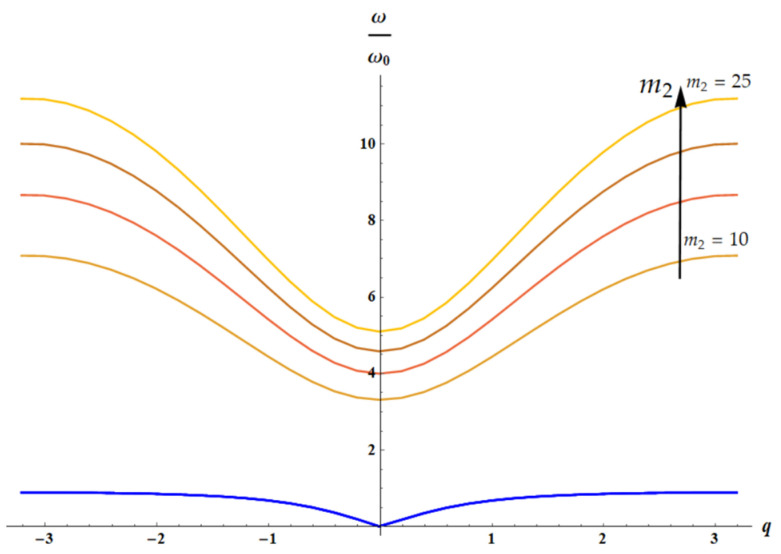
The solution of dispersion equation (Equation (18)) is depicted: acoustic (blue curves) and optical branches (from brown ($m_{2}=10$) to orange ($m_{2}=25$) curves), calculated for $m_{2}\in \left\{10,\;15,\;20,\;25\right\}$. The arrow indicates the direction of increase in parameter $m_{2}$ value. It is seen that dependence of acoustic mode on $m_{2}$ for $m_{2}\gg 1:\;m_{2}\in \left\{10,\;15,\;20,\;25\right\}$ is negligible.

It is clear that the solution of the dispersion equation (Figure 9) does not depend on temperature for $\frac{\omega (q)}{\omega_{0}}=\frac{\omega (q)}{\sqrt{\frac{k_{2}}{m_{2}}}}=\frac{\omega (q)}{\sqrt{\frac{T}{m_{2}}}}.$

**Figure 10:**
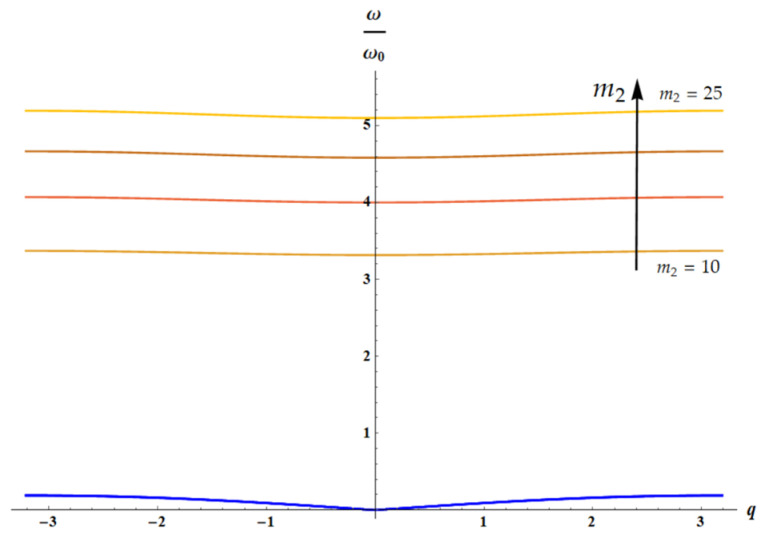
The solution of dispersion equation (Equation (17)) for $k_{1}=1,\;k_{2}=T$ is depicted: acoustic (blue curves) and optical branches (from brown ($m_{2}=10$) to orange ($m_{2}=25$) curves), calculated for $m_{2}\in \left\{10,\;15,\;20,\;25\right\}$. The arrow indicates the direction of increase in parameter $m_{2}$ value.

**Figure 11:**
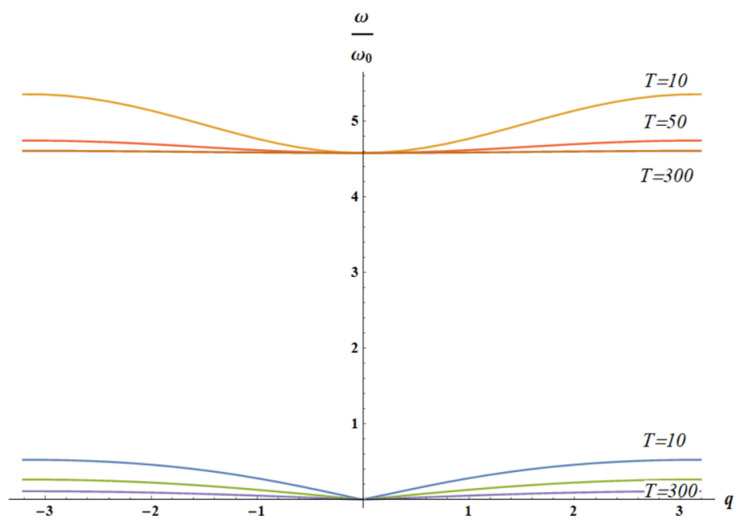
The solution of dispersion equation (Equation (17)) for $k_{1}=1,\;k_{2}=T$ is depicted. Here $m_{2}=20;$ $T\in \left\{10,\;50,\;300\right\}$. It is evident that with increasing temperature *T* both acoustic (blue, green and purple) and optical branches (orange, red and brown) depend weakly on the wave vector *q*.

Let us consider two cases: $k_{1}=1,\;k_{2}=T$ (only spring $k_{2}$ is entropic), shown in Figure 10, and $k_{1}=T,\;k_{2}=1$ (only spring $k_{1}$ is entropic), illustrated with Figure 11.

**Figure 12:**
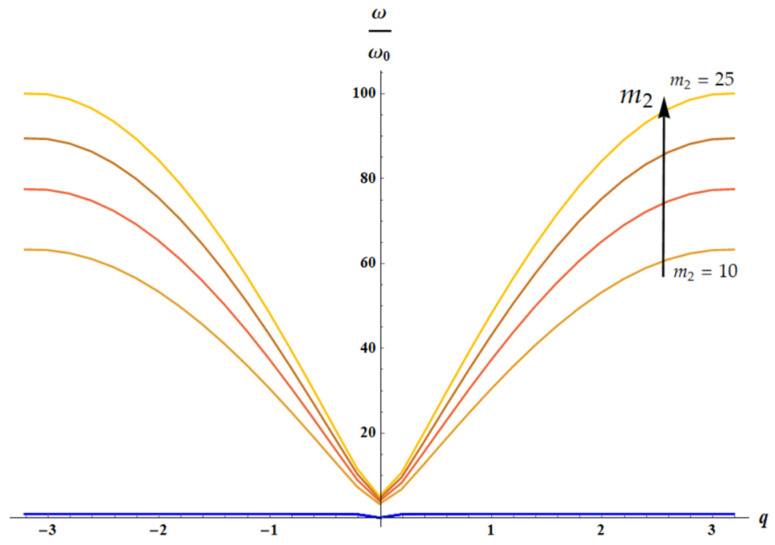
The solution of dispersion equation (Equation (17)) for $k_{1}=T,\;k_{2}=1$ is depicted: acoustic (blue curves) and optical branches (from brown ($m_{2}=10$) to orange ($m_{2}=25$) curves), calculated for $m_{2}\in \left\{10,\;15,\;20,\;25\right\}$. The arrow indicates the direction of increase in parameter $m_{2}$ value.

**Figure 13:**
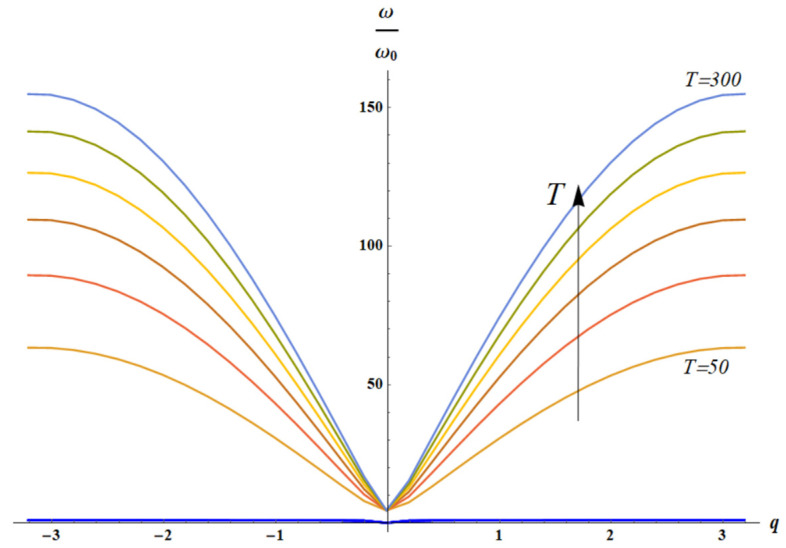
The solution of dispersion equation (Equation (17)) for $k_{1}=T,\;k_{2}=1$ is depicted. Here $m_{2}=20;$ temperature *T* changes from 50 (orange “optical” branch) to 300 (light blue “optical” curve), with a step of 50. Acoustic branch (blue curves) is barely dependent on temperature.

Figure 12 and Figure 13 illustrate the situation when the optical branch of the vibrations is strongly temperature-dependent, whereas the acoustic branch is slightly temperature-dependent.

## 4. Discussion

Resonances are ubiquitous in nature and engineering [37,38,39]. It is well known that the resonant phenomena may be temperature-dependent. The temperature dependence of the resonance frequency of the fundamental and four higher-order modes of a silicon dioxide micro-cantilever was established [40]. The temperature effects in resonant Raman spectroscopy were registered [41]. A temperature-dependent Raman resonant response in $UO_{2}$ was reported [42]. The temperature dependence of the Fano resonance discovered in infrared spectra of nano-diamonds was discussed [43]. We address the temperature-dependent resonant effects giving rise the phenomenon of the “negative effective mass effect” exerted on the intensive research of the last decade.

It is unnecessary to say that there is actually no negative mass [29,44,45]. The phenomenon of “negative effective mass” arises when we substitute the core–shell mechanical systems comprising a pair of masses (*M* and *m*) and the massless Hookean spring *k* by a single effective mass $m_{eff}$; that is to say that the internal mass *m* is hidden and its influence is expressed by the introduction of the mass $m_{eff}$ [21,22,23,29,44]. The negative effective mass represents the contra-intuitive situation of “anti-vibrations”, when the harmonic acceleration of the system is in an opposite direction to the sinusoidal applied force [21,22,23,28,29]. We considered the situation when the vibrations are driven by the temperature-dependent entropic forces inherent for natural and biological polymer systems [13,14,15,16,17,18,19,46]. The stiffness of the polymer spring is temperature-dependent, and the physical situation resembling the parametric resonance emerges when the temperature is varied [45]. Thus, the temperature-dependent effective mass emerges. The effect may be exemplified with polymer acoustic meta-materials [36,47]. The temperature-dependent effective mass is not a novel concept; it is broadly used in semiconductors [48]. However, in our analysis, the effect of temperature-dependent mass emerges from the entropic origin of the elastic force in resonant systems.

The engineering implementation of a suggested approach may be realized with rubbers or thermoplastic elastomers [14,15,16]. Negative density materials exploiting thermoplastic materials were already reported [49]. In particular, a negative mass system/waveguide based on styrene butadiene rubber was introduced [50]. These systems have the potential for temperature-dependent negative mass behavior.

## 5. Conclusions

We conclude that the effect of the temperature-dependent effective mass becomes possible in core–shell systems in which the spring connecting the core mass to the shell is driven by temperature-dependent entropic elasticity, such as that inherent for polymer materials. The core–shell system may be replaced with a single effective mass $m_{eff}\left(\omega ,T\right)$ exposed to the external harmonic force. When the frequency of the external force *ω* is fixed and the temperature of the core–shell system *T* is varied, the resonance becomes possible, and the harmonic acceleration of the shell may move in an opposite direction to the applied force. We demonstrate that the effective mass $m_{eff}\left(T\right)$ becomes negative when the temperature of the core–shell system approaches the critical temperature $T^{*}$ from below.

We also considered the chain/lattice built of core–shell units, in which entropic forces are acting. In this case, the effect of “negative density” is attainable through a variation in the temperature of the system. Again, the negative density $\rho_{eff}\left(T,\omega \right)$ becomes negative when the temperature of the core–shell system approaches the critical temperature $T^{*}$ from below. The critical temperature is defined by the parameters of polymer chain and frequency of the external force *ω*. We varied the parameters of the lattice built of the core–shell elements and connected with the elastic springs. An increase in the stiffness of the springs connecting the core–shell units leads to the sharpening of the resonance behavior of $\rho_{eff}(T)$ dependence. We also addressed the situation, and calculated the resonance curves, when we removed the temperature dependence of the elasticity of the “inner” and “outer” elastic elements one by one. In the low-temperature limit, the effective density $\rho_{eff}\left(T,\;\omega \right)$ almost does not change with the ratio of the elastic constants of the “internal” and “external springs” appearing in the lattice. Optical and acoustical branches of vibrations were calculated. The effect may be demonstrated experimentally with polymer meta-materials.

## Data Availability

The original contributions presented in this study are included in the article. Further inquiries can be directed to the corresponding authors.
